# Supplementary material for: Brain asymmetries from mid- to late life and hemispheric brain age
Source: Nat Commun. 2024 Feb 1;15:956. doi: 10.1038/s41467-024-45282-3 (PMC10834516; doi:10.1038/s41467-024-45282-3)
Supplement: Supplementary file 1 — Supplementary Information [file 41467_2024_45282_MOESM1_ESM.pdf]

SUPPLEMENTARY INFORMATION

Supplementary information to the article "Brain asymmetries from mid- to late-life and hemispheric brain age", Korbmacher et al., 2023

SUPPLEMENTARY TABLES

Supplementary Table 1. Tuned hyperparameters for brain age models considering both sexes together

Overview of the tuned hyperparameters for each of the used brain age models considering both sexes together. Source data are provided in Source Data file 5. Training

**Modality**	**Hemisphere**	**Learning Rate**	**Maximum Depth**	**Number of Trees**
Multimodal	Both	0.1	8	140
Multimodal	Left	0.05	7	180
Multimodal	Right	0.1	8	140
dMRI	Both	0.1	6	100
dMRI	Left	0.1	4	180
dMRI	Right	0.1	5	180
T_1w	Both	0.1	5	140
T_1w	Left	0.1	6	140
T_1w	Right	0.1	6	180

samples correspond with the sample sizes of all participants, also specified in the Methods section under participants): $N_{diffusion\ brain\ age} = 39,637$, $N_{T_1-derived\ brain\ age} = 48,040$, $N_{multimodal\ brain\ age} = 39,507$.

Supplementary Table 2. Most age-sensitive regional features using non-linear models

F-values were Bonferroni-corrected and all p-values are $p < 0.001$. $N_{diffusion\ metrics} = 39,637$, $N_{T_1-derived\ metrics} = 48,040$. Source data are provided in Source Data file 6.

T_1 Metric	Deviance	F	dMRI Metric	Deviance	F
superior temporal thickness (lh)	587304.16	4188.91	DKI - AK anterior limb of the internal capsule (rh)	644106.76	5170.95
hippocampus volume (rh)	576250.86	4101.39	DTI - RD fornix striaterminalis (rh)	627313.71	4981.99
thickness (lh)	576355.10	4082.87	DTI - FA anterior corona radiata (lh)	571637.61	4390.91
inferiorparietal thickness (lh)	569468.00	4041.74	DTI - FA inferior fronto-occipital fasciculus (lh)	568799.27	4366.64
hippocampus volume (lh)	565456.80	4006.59	BRIA - microRD anterior thalamic radiation (rh)	561902.12	4295.66
thickness (rh)	562548.97	3965.59	WMTI - radEAD anterior coronaradiata (rh)	433925.45	4281.90
inferior lateral ventricle volume (lh)	544864.71	3836.12	BRIA - microFA fornix striaterminalis (rh)	557084.22	4247.55
inferior lateral ventricle volume (rh)	539066.94	3786.01	DTI - FA fornix striaterminalis (rh)	545272.55	4125.27
superior temporal thickness (rh)	522564.64	3603.62	BRIA - microRD fornix striaterminalis (rh)	539180.21	4070.72
lateral ventricle volume (lh)	513713.08	3567.34	BRIA - microADC anterior thalamic radiation (rh)	536979.62	4050.30

Supplementary Table 3. Most age-sensitive regional features using linear models

$N_{diffusion\ metrics} = 39,637$, $N_{T_1-derived\ metrics} = 48,040$. Source data are provided in Source Data file 7.

T_1 Metric	Sum of Squares	F	dMRI Metric	Sum of Squares	F
superior temporal thickness (lh)	582215.80	12516.42	DTI - RD fornix striaterminalis (rh)	568838.72	13114.24
thickness (lh)	571936.88	12239.14	DTI - FA anterior coronaradiata (lh)	554045.66	12664.20
hippocampus volume (rh)	564806.62	12048.28	DTI - FA inferior fronto-occipital fasciculus (lh)	527205.85	11866.98
inferiorparietal thickness (rh)	559834.17	11915.90	DTI - FA fornix striaterminalis (rh)	526713.03	11852.57
thickness (rh)	557696.94	11859.18	DTI - RD anterior coronaradiata (lh)	504149.35	11201.30
hippocampus volume (lh)	554478.02	11773.95	DTI - RD anterior coronaradiata (rh)	500047.51	11084.67
superior temporal thickness (rh)	519361.12	10859.68	DTI - FA anterior coronaradiata (rh)	481860.37	10573.93
thalamus volume (rh)	470220.77	9626.25	BRIA - microRD anterior thalamic radiation (rh)	480010.76	10522.57
cortex volume (lh)	455643.18	9270.23	DTI - RD inferior fronto-occipital fasciculus (lh)	471710.65	10293.35
amygdala (lh)	454268.29	9236.88	DTI - FA inferior fronto-occipital fasciculus (rh)	470227.52	10252.62

Supplementary Table 4. Global metrics' age sensitivity using linear models

LRTs outcomes testing global metrics' age sensitivity using linear models (Eqs. 2 & 4), with p-values being Bonferroni-corrected for multiple comparison. Acronyms lh and rh refer to mean left and right hemisphere, respectively. $N_{diffusion\ metrics} = 39,637$, $N_{T_1-derived\ metrics} = 48,040$. Source data are provided in Source Data file 8.

**Metric**	**Sum of Squares**	**F**	**p**	**Metric**	**Sum of Squares**	**F**	**p**
BRIA vintra (lh)	-14143.51	256.78	<.001	*DTI MD (rh)*	-294821.39	6256.88	<.001
BRIA vintra (rh)	-13492.91	244.88	<.001	*DTI FA (lh)*	-294054.08	6237.71	<.001
BRIA vextra (lh)	-8868.68	160.58	<.001	*DTI FA (rh)*	-290846.08	6157.77	<.001
BRIA vextra (rh)	-8247.91	149.29	<.001	*SMT FA (lh)*	-96237.02	1824.32	<.001
BRIA vcsf (lh)	-12339.56	223.82	<.001	*SMT FA (rh)*	-88924.97	1679.10	<.001
BRIA vcsf (rh)	-11691.20	211.99	<.001	*SMT MD (lh)*	-145717.99	2837.80	<.001
BRIA micrord (lh)	-110749.44	2115.93	<.001	*SMT MD (rh)*	-138236.90	2681.03	<.001
BRIA micrord (rh)	-112757.19	2156.64	<.001	*SMT trans (lh)*	-236947.06	4859.34	<.001
BRIA microfa (lh)	-7389.49	133.69	<.001	*SMT trans (rh)*	-230976.33	4720.50	<.001
BRIA microfa (rh)	-7660.08	138.61	<.001	*SMT long (lh)*	-233251.22	4773.28	<.001
BRIA microax (lh)	-20330.95	370.29	<.001	*SMT long (rh)*	-221802.60	4509.03	<.001
BRIA microax (rh)	-19217.81	349.82	<.001	*SMTmc d (lh)*	-12811.82	232.44	<.001
BRIA microadc (lh)	-244852.70	5044.67	<.001	*SMTmc d (rh)*	-15325.44	278.40	<.001
BRIA microadc (rh)	-242965.40	5000.27	<.001	*SMTmc extramd (lh)*	-234164.26	4794.51	<.001
BRIA dradextra (lh)	-0.87	0.02	1.00	*SMTmc extramd (rh)*	-221755.78	4507.96	<.001
BRIA dradextra (rh)	-0.56	0.01	1.00	*SMTmc extratrans (lh)*	-269921.51	5643.84	<.001
BRIA daxintra (lh)	-45776.98	844.85	<.001	*SMTmc extratrans (rh)*	-251971.27	5213.02	<.001
BRIA daxintra (rh)	-32572.59	597.02	<.001	*SMTmc intra (lh)*	-162286.59	3189.66	<.001
BRIA daxextra (lh)	-33941.70	622.56	<.001	*SMTmc intra (rh)*	-138122.05	2678.64	<.001
BRIA daxextra (rh)	-29058.51	531.64	<.001	*WMTI awf (lh)*	-216212.24	4381.26	<.001
DKI AK (lh)	-98394.96	1867.39	<.001	*WMTI awf (rh)*	-198966.98	3992.24	<.001
DKI AK (rh)	-107687.41	2054.02	<.001	*WMTI radead (lh)*	-538.93	9.72	0.11
DKI RK (lh)	-134762.36	2608.66	<.001	*WMTI radead (rh)*	-1786.25	32.22	<.001
DKI RK (rh)	-117109.00	2245.17	<.001	*WMTI axead (lh)*	-15537.30	282.28	<.001
DKI MK (lh)	-166559.26	3281.46	<.001	*WMTI axead (rh)*	-140593.59	2730.28	<.001
DKI MK (rh)	-146629.45	2856.99	<.001	*T1 (lh) thickness*	-361976.02	7460.14	<.001
DTI AD (lh)	-6414.25	115.99	<.001	*T1 (rh) thickness*	-337720.79	6873.27	<.001
DTI AD (rh)	-32682.00	599.06	<.001	*T1 (lh) area*	-131984.99	2428.71	<.001
DTI RD (lh)	-103169.79	1963.06	<.001	*T1 (rh) area*	-115500.16	2109.17	<.001
DTI RD (rh)	-98654.94	1872.59	<.001	*T1 (lh) volume*	-366138.06	7562.34	<.001
DTI MD (lh)	-296264.43	6292.97	<.001	*T1 (rh) volume*	-351072.41	7194.50	<.001

Supplementary Table 5. Global metrics' age sensitivity using non-linear models

LRTs outcomes testing global metrics' age sensitivity using generalized additive models (Eqs. (3,4)), with p-values being Bonferroni-corrected for multiple comparison. Acronyms lh and rh refer to mean left and right hemisphere, respectively. $N_{diffusion\ metrics} = 39,637$, $N_{T_1-derived\ metrics} = 48,040$. Source data are provided in Source Data file 9.

Metric	Deviance	F	p	Metric	Deviance	F	p
BRIA vintra (lh)	298222.11	1980.42	<.001	*DTI MD (rh)*	420292.82	2975.29	<.001
BRIA vintra (rh)	263814.46	1721.75	<.001	*DTI FA (lh)*	454980.03	3284.21	<.001
BRIA vextra (lh)	99954.26	601.06	<.001	*DTI FA (rh)*	437831.65	3130.91	<.001
BRIA vextra (rh)	68415.48	404.41	<.001	*SMT FA (lh)*	231126.97	1481.17	<.001
BRIA vcsf (lh)	389707.46	2715.85	<.001	*SMT FA (rh)*	212502.18	1350	<.001
BRIA vcsf (rh)	395906.41	2768.18	<.001	*SMT MD (lh)*	338666.06	2295.03	<.001
BRIA micrord (lh)	489922	3605.08	<.001	*SMT MD (rh)*	329913.59	2225.14	<.001
BRIA micrord (rh)	482669.31	3537.29	<.001	*SMT trans (lh)*	325557.05	2188.8	<.001
BRIA microfa (lh)	468131.01	3399.04	<.001	*SMT trans (rh)*	309770.56	2066.61	<.001
BRIA microfa (rh)	441798.75	3161.43	<.001	*SMT long (lh)*	239399.25	1543.22	<.001
BRIA microax (lh)	123284.12	747.18	<.001	*SMT long (rh)*	220310.81	1406.65	<.001
BRIA microax (rh)	122353.86	741.87	<.001	*SMTmc d (lh)*	17581.83	100.96	<.001
BRIA microadc (lh)	442217.61	3169.41	<.001	*SMTmc d (rh)*	18705.17	107	<.001
BRIA microadc (rh)	433573.24	3092.76	<.001	*SMTmc extramd (lh)*	375805.34	2598.83	<.001
BRIA dradextra (lh)	265199.9	1732.72	<.001	*SMTmc extramd (rh)*	350591.17	2392.53	<.001
BRIA dradextra (rh)	259410.42	1690.27	1.00	*SMTmc extratrans (lh)*	381698.57	2646.36	<.001
BRIA daxintra (lh)	227477.58	1459.06	<.001	*SMTmc extratrans (rh)*	357451.71	2446.62	<.001
BRIA daxintra (rh)	221619.72	1417.52	<.001	*SMTmc intra (lh)*	230534.22	1477.89	<.001
BRIA daxextra (lh)	269452.37	1764.08	<.001	*SMTmc intra (rh)*	196608	1238.41	<.001
BRIA daxextra (rh)	265820.53	1737.25	<.001	*WMTI awf (lh)*	294396.47	1946.39	<.001
DKI AK (lh)	248201.74	1607.19	<.001	*WMTI awf (rh)*	271308.81	1773.47	<.001
DKI AK (rh)	277452.37	1822.07	<.001	*WMTI radead (lh)*	356837.69	2444	<.001
DKI RK (lh)	248246.37	1606.74	<.001	*WMTI radead (rh)*	347896.75	2371.95	<.001
DKI RK (rh)	214591.89	1365.38	<.001	*WMTI axead (lh)*	22893.57	133.33	<.001
DKI MK (lh)	225899.98	1446.06	<.001	*WMTI axead (rh)*	30036.73	175.61	<.001
DKI MK (rh)	190685.71	1195.84	<.001	*T1 (lh) thickness*	363679.29	2447.65	<.001
DTI AD (lh)	91486.87	545.48	<.001	*T1 (rh) thickness*	339637.31	2256.41	<.001
DTI AD (rh)	63150.51	374.43	<.001	*T1 (lh) area*	132330.67	818.92	<.001
DTI RD (lh)	492407	3628.18	<.001	*T1 (rh) area*	115697.02	777.46	<.001
DTI RD (rh)	481438.43	3525.6	<.001	*T1 (lh) volume*	366575.45	2414.26	<.001
DTI MD (lh)	425442.7	3020.18	<.001	*T1 (rh) volume*	351519.39	2312.27	<.001

Supplementary Table 6. Differences of T_1-weighted and dMRI features between hemispheres by sex

The table shows the ten largest regional differences between left and right hemispheres' T_1-weighted and dMRI data indicated by effect size (Cohen's d) indicated by paired samples t-tests (two-sided) and presented separately for males: $N_{diffusion\ metrics} = 18,950$, $N_{T_1-derived\ metrics} = 23,059$ and females: $N_{diffusion\ metrics} = 20,683$, $N_{T_1-derived\ metrics} = 24,981$. All Bonferroni corrected $p < .001$. SLFT = Superior longitudinal fasciculus (temporal part), ILF = Inferior longitudinal fasciculus. Source data are provided in Source Data file 10.

**diffusion MRI**			
**Feature**	**Cohen's** d_{males}	**Feature**	**Cohen's** $d_{females}$
DTI - FA ILF	3.44	DTI - FA ILF	3.91
DTI - AD SLFT	2.09	DTI - AD SLFT	2.40
WMTI - axEAD SLFT	2.01	SMTmc - diff SLFT	2.06
DTI - FA cingulate gyrus	1.93	SMT - long SLFT	2.04
DKI - RK cingulate gyrus	1.90	DTI - FA cingulate gyrus	1.98
WMTI - AWF cingulate gyrus	1.83	SMTmc - extratrans cerebral peduncle	1.96
DTI - AD ILF	1.81	SMTmc - extraMD SLFT	1.93
DTI - FA superior frontooccipital fasciculus	1.77	BRIA - microAX SLFT	1.92
DKI - RK SLFT	1.75	DKI - RK SLFT	1.91
SMTmc - extratrans cerebral peduncle	1.74	SMTmc - intra cingulate gyrus	1.89
**T_1-weighted MRI**			
**Feature**	**Cohen's** d_{males}	**Feature**	**Cohen's** $d_{females}$
frontal pole area	1.82	transverse temporal area	1.89
pars orbitalis area	1.78	frontal pole area	1.73
transverse temporal area	1.77	pars orbitalis area	1.72
inferior parietal area	1.71	inferior parietal area	1.72
inferior parietal volume	1.62	inferior parietal volume	1.64
frontal pole volume	1.58	frontal pole volume	1.54
thalamus volume	1.40	middle temporal area	1.42
middle temporal area	1.31	transverse temporal volume	1.38
transverse temporal volume	1.29	thalamus volume	1.34
pars orbitalis volume	1.27	pars orbitalis volume	1.29

Supplementary Table 7. Most age-sensitive regional T_1- and diffusion-weighted features using *non*-linear models *by sex*

The table shows the ten largest regional differences between left and right hemispheres' T_1-weighted and dMRI data indicated by F from LRTs comparing a baseline model (Eq. 4) to the GAM (Eq. 3) presented separately for males: $N_{diffusion\ metrics} = 18,950$, $N_{T_1-derived\ metrics} = 23,059$ and females: $N_{diffusion\ metrics} = 20,683$, $N_{T_1-derived\ metrics} = 24,981$. All Bonferroni corrected $p < .001$. ATR = Anterior thalamic radiation, IFOF = inferior fronto-occipital fasciculus. Source data are provided in Source Data file 11.

Males

T_1 Metric	Deviance	F	dMRI Metric	Deviance	F
Hippocampus volume (rh)	325396.71	2327.14	DTI - RD fornix striaterminalis (rh)	321483.12	2474.87
Inferior lateral ventricle volume (lh)	315630.20	2242.52	DKI - AK Anteriorlimbofinternalcapsule (rh)	315123.70	2406.94
Hippocampus volume (lh)	314178.29	2222.80	DTI - FA fornix striaterminalis (rh)	287920.97	2127.34
Lateral ventricle volume (rh)	294791.55	2055.25	DTI - FA IFOF (lh)	286229.79	2114.11
Superior temporal thickness (lh)	288883.19	1973.34	BRIA - micro Rd ATR (rh)	285675.67	2109.47
Thickness (lh)	286653.65	1944.76	DTI - FA Anteriorcoronaradiata (lh)	285098.32	2099.07
Thickness (rh)	285659.63	1943.02	BRIA - micro FA Fornix Striaterminalis (rh)	280978.76	2062.43
Lateral ventricle volume (lh)	280759.99	1932.22	BRIA - micro Rd ATR (lh)	268901.80	1946.45
Bankssts thickness (lh)	111534.08	1927.07	DTI - RD ATR (rh)	268857.43	1944.99
Rostral middle frontal volume (rh)	112544.65	1887.40	DTI - RD ATR (lh)	268408.62	1942.57

Females

T_1 Metric	Deviance	F	dMRI Metric	Deviance	F
Superior temporal thickness (lh)	298436.46	2186.28	DKI - AK Anteriorlimbofinternalcapsule (rh)	328299.92	2756.78
Inferior parietal thickness (rh)	294083.30	2157.56	DTI - RD Fornix Striaterminalis (rh)	309568.49	2539.12
Thickness (lh)	289859.17	2098.94	DTI - FA Anteriorcoronaradiata (lh)	287115.30	2288.94
Thickness (rh)	277328.73	1988.89	DTI - FA IFOF (lh)	282230.37	2243.21
Superiortemporal thickness (rh)	268345.92	1902.38	BRIA - micro FA Fornix Striaterminalis (rh)	279454.04	2213.63
Hippocampus volume (lh)	256888.62	1827.15	BRIA - micro Rd Fornix Striaterminalis (rh)	279158.45	2213.20
Hippocampus volume (rh)	256386.19	1820.25	BRIA - micro Rd ATR (rh)	279221.52	2209.87
Lateral ventricle volume (rh)	247973.59	1755.77	DTI - RD ATR (lh)	278213.99	2202.86
Lateral ventricle volume (lh)	237509.49	1666.32	DTI - RD Anteriorcoronaradiata (lh)	277873.07	2197.90
Supramarginal thickness (rh)	235411.09	1632.94	BRIA - micro Rd ATR (lh)	274318.30	2160.44

Supplementary Table 8. Most age-sensitive regional T_1- and diffusion-weighted features using linear models *by sex*

The table shows the ten largest regional differences between left and right hemispheres' T_1-weighted and dMRI data indicated by F from LRTs comparing a baseline model (Eq. 4) to the linear model (Eq. 2) presented separately for males: $N_{diffusion\ metrics} = 18,950$, $N_{T_1-derived\ metrics} = 23,059$ and females: $N_{diffusion\ metrics} = 20,683$, $N_{T_1-derived\ metrics} = 24,981$. All Bonferroni corrected $p < .001$. ATR = Anterior thalamic radiation, SLFT = superior longitudinal fasciculus (temporal part), IFOF = inferior fronto-occipital fasciculus. Source data are provided in Source Data file 12.

Males

T_1 Metric	SS	F	dMRI Metric	SS	F
Hippocampus volume (rh)	316544.24	6766.75	DTI - RD fornix striaterminalis (rh)	286594.17	6364.49
Hippocampus volume (lh)	306351.25	6487.54	DTI - FA fornix striaterminalis (rh)	276467.17	6067.57
Superior temporal thickness (lh)	286384.87	5955.49	DTI - FA anterior corona radiata (lh)	274955.01	6023.83
Thickness (lh)	284626.62	5909.55	DTI - FA IFOF (lh)	263814.28	5706.24
Thickness (rh)	283428.75	5878.34	DTI - RD anterior corona radiata (lh)	246467.64	5227.51
Inferior parietal thickness (rh)	271677.80	5575.68	DTI - RD anterior corona radiata (rh)	242227.42	5113.30
Inferior lateral ventricle volume (lh)	254969.32	5156.08	DTI - FA anterior corona radiata (rh)	238605.78	5016.60
Superior temporal thickness (rh)	252923.59	5105.55	DTI - FA IFOF (rh)	233533.32	4882.47
Thalamus volume (rh)	247813.43	4980.11	BRIA - microRD ATR (rh)	232131.22	4845.66
Amygdala volume (lh)	243131.64	4866.16	DTI - RD IFOF (lh)	230505.57	4803.12

T_1 Metric	SS	F	dMRI Metric	SS	F
Superior temporal thickness (lh)	295682.20	6561.30	DTI - RD fornix striaterminalis (rh)	282234.33	6743.14
Inferior parietal thickness (rh)	288649.89	6365.48	DTI - FA anterior corona radiata (lh)	279013.72	6641.48
Thickness (lh)	287657.68	6338.05	DTI - FA IFOF (lh)	263549.29	6163.65
Thickness (rh)	274795.27	5986.72	DTI - RD anterior corona radiata (rh)	258369.12	6007.30
Superior temporal thickness (rh)	266558.45	5765.84	DTI - RD anterior corona radiata (lh)	257953.77	5994.85
Hippocampus volume (rh)	248481.56	5291.98	DTI - FA fornix striaterminalis (rh)	251128.24	5791.79
Hippocampus volume (lh)	248438.59	5290.87	BRIA - microRD ATR (rh)	249535.85	5744.86
Supramarginal thickness (rh)	232265.05	4879.14	BRIA - microRD ATR (lh)	243660.64	5573.15
Supramarginal thickness (lh)	225512.01	4710.53	DTI - FA anterior corona radiata (rh)	243324.99	5563.40
Precuneus thickness (rh)	223530.22	4661.41	DTI - RD IFOF (lh)	241625.80	5514.19

Supplementary Table 9. Description of white matter features by diffusion approaches.

**Diffusion Approach**	**Metrics**
Bayesian Rotationally Invariant Approach (BRIA) [1]	intra-axonal axial diffusivity (DAX intra)
	extra-axonal radial diffusivity (DRAD extra)
	microscopic fractional anisotropy (micro FA)
	extra-axonal axial diffusivity (DAX extra)
	intra-axonal water fraction (V intra)
	extra-axonal water fraction (V extra)
	cerebrospinal fluid fraction (vCSF)
	microscopical axial diffusivity (micro AX)
	microscopic radial diffusivity (micro RD)
	microscopical apparent diffusion coefficient (micro ADC)
Diffusion Kurtosis Imaging (DKI) [2, 3]	mean kurtosis (MK)
	radial kurtosis (RK)
	axial kurtosis (AK)
Diffusion Tensor Imaging (DTI) [4]	fractional anisotropy (FA)
	axial diffusivity (AD)
	mean diffusivity (MD)
	radial diffusivity (RD)
Spherical Mean Technique (SMT) [5]	fractional anisotropy (SMT FA)
	mean diffusivity (SMT md)
	transverse diffusion coefficient (SMT trans)
	longitudinal diffusion coefficient (SMT long)
Multi-compartment Spherical Mean Technique (SMTmc) [6]	extra-neurite microscopic mean diffusivity (SMTmc extra md)
	extra-neurite transverse microscopic diffusivity (SMTmc extra trans)
	mc SMTdiffusion coefficient (SMT mcd)
	intra-neurite volume fraction (SMTmc intra)
White Matter Tract Integrity (WMTI) [3]	axonal water fraction (AWF)
	radial extra-axonal diffusivity (radEAD)
	axial extra-axonal diffusivity (axEAD)

Supplementary Table 10. Differences of T_1w and dMRI features between hemispheres

The table shows the ten largest regional differences between left and right hemispheres' T_1-weighted ($N = 48,040$) and diffusion MRI data ($N = 39,637$) indicated by effect size (Cohen's d) indicated by paired samples t-tests (two-sided). SLFT = Superior longitudinal fasciculus (temporal part), ILF = Inferior longitudinal fasciculus. Bonferroni-adjusted p-values were $p < 2 \times 10^{-308}$. Source data are provided in Source Data file 13.

**T_1-weighted MRI**			**diffusion MRI**		
**Feature**	**T-value**	**Cohen's d**	**Feature**	**T-value**	**Cohen's d**
Transverse temporal area	397.45	1.81	DTI - FA ILF	725.48	3.64
Frontal pole area	-386.34	1.76	DTI - AD SLFT	-444.89	2.23
Pars orbitalis area	-380.71	1.74	DTI - FA cingulate gyrus	388.09	1.95
Inferior parietal area	-368.85	1.68	DKI - RK cingulate gyrus	375.36	1.89
Inferior parietal volume	-352.95	1.61	SMTmc - diff SLFT	-369.19	1.85
Frontal pole volume	-340.08	1.55	SMTmc - extratrans cerebral peduncle	-367.31	1.84
Middletemporal area	-297.79	1.36	DKI - RK SLFT	-364.52	1.83
Thalamus Proper	296.93	1.35	WMTI - AWF cingulate gyrus	364.46	1.83
Transverse temporal volume	292.04	1.33	SMT - long SLFT	-359.08	1.80
Pars orbitalis volume	-280.74	1.28	DTI - AD ILF	353.43	1.78

Supplementary Table 11. Permutation feature importance for multimodal, T_1-weighted, and dMRI features between hemispheres considering *both sexes together*

Permutation feature importance shows the contribution of each feature $R^2 \pm SD$ (standard deviation) to the (brain) age predictions using multimodal ($N = 39,507$), T_1-weighted ($N = 48,040$), and dMRI models ($N = 39,637$) of each hemisphere on their own and both hemispheres together. Retroen. = Retrolenticular, l.o.int.caps. = limb of the internal capsule, cerebell.ped. = cerebellar peduncle. ATR = anterior thalamic radiation, CST = corticospinal tract, IFOF = inferior fronto-occipital fasciculus, SLF = superior longitudinal fasciculus.

**Multimodal MRI**					
**Both hemispheres**		**Left hemisphere**		**Right hemisphere**	
DKI – AK Anterior l.o.int.caps. (rh)	0.083 ± 0.0010	DTI – AK Anterior l.o.int.caps.	0.058 ± 0.0007	DKI – AK Anterior l.o.int.caps.	0.096 ± 0.0006
DTI – RD Fornix Striaterminalis (rh)	0.049 ± 0.0006	DTI – FA Superior cerebell.ped.	0.031 ± 0.0004	DTI – RD Fornix Striaterminalis	0.033 ± 0.0004
Cortex volume (lh)	0.018 ± 0.0004	Cerebellum WM volume	0.023 ± 0.0004	DTI – FA Superior cerebell.ped.	0.03 ± 0.0004
DTI – FA Cerebral peduncle (lh)	0.015 ± 0.0003	Inferior Lateral Ventricle volume	0.022 ± 0.0003	Cerebellum WM volume	0.024 ± 0.0003
DKI – AK Anterior l.o.int.caps. (lh)	0.013 ± 0.0002	Thalamus volume	0.019 ± 0.0003	Thalamus volume	0.02 ± 0.0002
DTI – FA Superior cerebell.ped. (lh)	0.011 ± 0.0002	DKI – RK Fornix-stria terminalis	0.019 ± 0.0003	BRIA – vCSF external capsule	0.018 ± 0.0003
ROI 3a area (rh)	0.01 ± 0.0002	Putamen volume	0.019 ± 0.0003	Hippocampus volume	0.017 ± 0.0003
ROI 3a area (lh)	0.01 ± 0.0002	BRIA – vCSF External capsule	0.019 ± 0.0003	Lateral ventricle volume	0.017 ± 0.0001
BRIA – vCSF External capsule (lh)	0.01 ± 0.0002	Lateral Ventricle volume	0.017 ± 0.0003	DKI – RK Posterior l.o.int.caps.	0.016 ± 0.0002
DTI – FA Superior cerebell.ped. (rh)	0.008 ± 0.0002	WMTI – AWF Superior cerebell.ped.	0.017 ± 0.0003	WMTI – AWF Retroen. l.o.int.caps.	0.014 ± 0.0002
**diffusion-weighted MRI**					
**Both hemispheres**		**Left hemisphere**		**Right hemisphere**	
DKI – AK Anterior l.o.int.caps. (rh)	0.103 ± 0.0012	DKI – AK Anterior l.o.int.caps.	0.095 ± 0.0013	DKI – AK Anterior l.o.int.caps.	0.126 ± 0.0014
DTI – RD Fornix Striaterminalis (rh)	0.061 ± 0.0009	BRIA – vCSF ATR	0.025 ± 0.0007	DTI – RD Fornix Stria terminalis	0.086 ± 0.0010
DTI – FA Cerebral peduncle (lh)	0.018 ± 0.0005	DKI – RK Fornix-stria terminalis	0.023 ± 0.0005	DTI – FA Superior cerebell.ped.	0.018 ± 0.0004
DTI – FA Anterior corona radiata (lh)	0.012 ± 0.0003	DTI – FA Fornix-stria terminalis	0.022 ± 0.0007	DKI – AK PTR	0.018 ± 0.0004
DKI – AK Anterior l.o.int.caps. (lh)	0.011 ± 0.0003	DTI – FA Cerebral peduncle	0.022 ± 0.0006	BRIA – vCSF SLF	0.017 ± 0.0004
DTI – FA Superior cerebell.ped. (lh)	0.010 ± 0.0003	DTI – FA Anterior corona radiata	0.022 ± 0.0005	DKI – AK Superio rcerebell.ped.	0.015 ± 0.0004
DTI – AD Superior l.o.int.caps. (lh)	0.010 ± 0.0003	WMTI – AWF Retroen. l.o.int.caps.	0.02 ± 0.0005	WMTI – AWF Retroen. l.o.int.caps.	0.014 ± 0.0003
DTI – AD Posterior l.o.int.caps. (lh)	0.010 ± 0.0003	DTI – FA IFOF	0.018 ± 0.0005	DTI – AD CST	0.013 ± 0.0004
DTI – AD CST (lh)	0.009 ± 0.0003	DKI – AK Superior frontooccipital fasciculus	0.017 ± 0.0006	BRIA – vCSF ATR	0.011 ± 0.0004
WMTI – AWF Retroen. l.o.int.caps. (lh)	0.009 ± 0.0002	DTI – FA Superior cerebell.ped.	0.016 ± 0.0005	DKI – RK Posterior l.o.int.caps.	0.01 ± 0.0002
**T1-weighted MRI**					
**Both hemispheres**		**Left hemisphere**		**Right hemisphere**	
Cortex volume (lh)	0.041 ± 0.0008	Lateral ventricle volume	0.071 ± 0.0011	Lateral ventricle volume	0.115 ± 0.0012
ROI PreS area (lh)	0.018 ± 0.0004	Inf.Lat.Vent volume	0.065 ± 0.0008	Inferiorparietal thickness	0.037 ± 0.0005
ROI 3a area (rh)	0.014 ± 0.0004	Superior temporal thickness	0.034 ± 0.0007	Superiortemporal thickness	0.036 ± 0.0006
Mean thickness (lh)	0.014 ± 0.0004	Insula volume	0.03 ± 0.0006	Inf.Lat.Vent volume	0.035 ± 0.0006
ROI Pol1 volume (rh)	0.013 ± 0.0005	Putamen volume	0.028 ± 0.0006	Inferior temporal area	0.029 ± 0.0005
ROI H area (lh)	0.011 ± 0.0003	Mean thickness	0.023 ± 0.0005	Thalamus volume	0.029 ± 0.0006
ROI PI thickness (lh)	0.011 ± 0.0004	Cerebellum WM volume	0.022 ± 0.0005	Cerebellum WM volume	0.028 ± 0.0005
ROI 52 area (lh)	0.011 ± 0.0003	Thalamus volume	0.02 ± 0.0005	Insula volume	0.028 ± 0.0006
ROI 3a area (lh)	0.009 ± 0.0003	Temporal pole volume	0.019 ± 0.0005	Superior frontal thickness	0.025 ± 0.0004
ROI H thickness (lh)	0.009 ± 0.0003	Amygdala volume	0.016 ± 0.0003	Hippocampus volume	0.022 ± 0.0005

Supplementary Table 12. Permutation feature importance for multimodal, T_1-weighted, and dMRI features between hemispheres considering *males only*

Permutation feature importance shows the contribution of each feature $R^2 \pm SD$ (standard deviation) to the (brain) age predictions using multimodal ($N = 18,876$), T_1-weighted ($N = 23,059$), and dMRI models ($N = 18,950$) of each hemisphere on their own and both hemispheres together. Inf.Lat.Vent. = Inferior Lateral Ventricle, retroen. = Retrolenticular, l.o. inf. int.caps = limb of the inferior internal capsule, Ant. = anterior, l.o. inf. ext.caps. = limb of the external capsule, l.o.int.caps. = limb of the internal capsule, cerebell.ped. = cerebellar peduncle, SFF = superior frontooccipital fasciculus, ATR = anterior thalamic radiation, CST = corticospinal tract, IFOF = inferior fronto-occipital fasciculus, SLF = superior longitudinal fasciculus, UF = uncinate fasciculus, PTR = Posterior thalamic radiation.

**Multimodal MRI**					
**Both hemispheres**		**Left hemisphere**		**Right hemisphere**	
DKI - AK Ant. l.o. inf. int.caps (rh)	0.063 ± 0.0009	DKI – AK Ant. l.o.int.caps.	0.049 ± 0.0009	DKI – AK Ant. l.o.int.caps.	0.071 ± 0.0012
DTI - RD Fornix-Striaterminalis (rh)	0.023 ± 0.0005	DTI – FA Superior cerebell.ped.	0.025 ± 0.0007	DTI – RD Fornix Stria	0.033 ± 0.0009
Inf.Lat.Vent. volume (lh)	0.015 ± 0.0004	Inf.Lat.Vent volume	0.024 ± 0.0006	DTI – FA Superior cerebell.ped.	0.028 ± 0.0009
Putamen volume (lh)	0.010 ± 0.0002	Cerebellum WM volume	0.023 ± 0.0005	Lateral ventricle volume	0.016 ± 0.0006
Thalams volume (lh)	0.009 ± 0.0003	Thalamus volume	0.022 ± 0.0005	Thalamus volume	0.015 ± 0.0005
Thalams volume (rh)	0.009 ± 0.0003	Putamen volume	0.019 ± 0.0006	Cerebellum WM volume	0.014 ± 0.0004
Amygdala volume (lh)	0.009 ± 0.0003	WMTI – AWF Superior cerebell.ped.	0.016 ± 0.0004	Hippocampus volume	0.013 ± 0.0004
DTI - FA Superior cerebell.ped. (rh)	0.009 ± 0.0003	Lateral Ventricle	0.016 ± 0.0004	BRIA – vCSF external capsule	0.012 ± 0.0005
BRIA - vCSF External capsule (lh)	0.009 ± 0.0002	DKI – RK Fornix-stria terminalis	0.013 ± 0.0004	WMTI – AWF Superior cerebell.ped.	0.010 ± 0.0029
Cerebellum WM volume (rh)	0.009 ± 0.0002	Amygdala volume	0.012 ± 0.0005	DKI – RK Posterior l.o.int.caps.	0.010 ± 0.0029
**diffusion-weighted MRI**					
**Both hemispheres**		**Left hemisphere**		**Right hemisphere**	
DKI - AK Ant. l.o. inf. ext.caps. (rh)	0.097 ± 0.0015	DKI – AK Ant. l.o.int.caps.	0.100 ± 0.0020	DKI – AK Ant. l.o.int.caps.	0.123 ± 0.0021
DTI - RD Fornix-Striaterminalis (rh)	0.061 ± 0.0013	BRIA – vCSF ATR	0.028 ± 0.0009	DTI – RD Fornix Striaterminalis	0.078 ± 0.0015
DTI - FA Cerebral peduncle (lh)	0.022 ± 0.0007	DTI – FA Cerebral peduncle	0.028 ± 0.0008	DTI – FA Superior cerebell.ped.	0.018 ± 0.0005
DTI - FA Ant. corona radiata (lh)	0.019 ± 0.0006	DTI – FA IFOF	0.027 ± 0.0008	BRIA – vCSF ATR	0.017 ± 0.0006
DKI - AK Ant. l.o. inf. ext.caps. (lh)	0.015 ± 0.0005	DTI – FA Ant. corona radiata	0.023 ± 0.0007	DTI – AD CST	0.015 ± 0.0005
DTI - FA Superior cerebell.ped. (lh)	0.012 ± 0.0004	DKI – RK Fornix-stria terminalis	0.021 ± 0.0006	DKI – AK PTR	0.013 ± 0.0004
BRIA - vextra SLF (rh)	0.011 ± 0.0004	DTI – FA Fornix-stria terminalis	0.021 ± 0.0008	WMTI – AWF retroen. int.caps.	0.012 ± 0.0004
DTI - FA IFOF (lh)	0.010 ± 0.0003	DKI – AK SFF	0.021 ± 0.0007	BRIA – vextra SLF	0.012 ± 0.0005
DKI - AK SFF (rh)	0.010 ± 0.0004	DTI – FA Superior cerebell.ped.	0.017 ± 0.0007	DKI – AK UF	0.011 ± 0.0005
BRIA - vCSF External capsule (lh)	0.009 ± 0.0002	WMTI – AWF retroen. l.o. int.caps.	0.016 ± 0.0006	DKI – AK ATR	0.011 ± 0.0003
**T_1-weighted MRI**					
**Both hemispheres**		**Left hemisphere**		**Right hemisphere**	
Inf.Lat.Vent (lh)	0.051 ± 0.0011	Inf.Lat.Vent volume	0.064 ± 0.0014	Lateral ventricle volume	0.107 ± 0.002
Lateral Ventricle (rh)	0.034 ± 0.0008	Lateral Ventricle	0.049 ± 0.0011	Inf.Lat.Vent volume	0.042 ± 0.0010
Thalamus volume (rh)	0.019 ± 0.0006	Putamen volume	0.036 ± 0.0011	Hippocampus volume	0.266 ± 0.0009
Superior temporal thickness (lh)	0.019 ± 0.0006	Insula volume	0.034 ± 0.0012	Thalamus volume	0.026 ± 0.0008
Putamen volume (lh)	0.017 ± 0.0006	Temporal pole volume	0.023 ± 0.0006	Isthmus cingulate thickness	0.022 ± 0.0005
Insula volume (lh)	0.017 ± 0.0007	Superior temporal thickness	0.023 ± 0.0006	Superior temporal thickness	0.021 ± 0.0007
Hippocampus volume (rh)	0.017 ± 0.0006	Amygdala volume	0.020 ± 0.0006	Temporal pole volume	0.020 ± 0.0007
Amygdala volume (rh)	0.017 ± 0.0006	Thalamus volume	0.018 ± 0.0006	Superior frontal thickness	0.019 ± 0.0005
Inf.Lat.Ventricle (rh)	0.016 ± 0.0007	Cerebellum WM volume	0.018 ± 0.0004	Cerebellum WM volume	0.019 ± 0.0006
Temporal pole volume (rh)	0.0162 ± 0.0007	Isthemus cingulate thickness	0.014 ± 0.0004	Insula volume	0.019 ± 0.0005

Supplementary Table 13. Permutation feature importance for multimodal, T_1-weighted, and dMRI features between hemispheres considering *females only*

Permutation feature importance shows the contribution of each feature $R^2 \pm SD$ (standard deviation) to the (brain) age predictions using multimodal ($N = 20,631$), T_1-weighted ($N = 24,981$), and dMRI models ($N = 20,683$) of each hemisphere on their own and both hemispheres together. Inf.Lat.Vent. = Inferior Lateral Ventricle, retroen. = Retrolenticular, l.o. inf. int.caps = limb of the inferior internal capsule, Ant. = anterior, l.o. inf. ext.caps. = limb of the external capsule, l.o.int.caps. = limb of the internal capsule, cerebell.ped. = cerebellar peduncle, SFF = superior frontooccipital fasciculus, ATR = anterior thalamic radiation, CST = corticospinal tract, IFOF = inferior fronto-occipital fasciculus, SLF = superior longitudinal fasciculus, UF = uncinate fasciculus, PTR = Posterior thalamic radiation.

		**Multimodal MRI**			
**Both hemispheres**		**Left hemisphere**		**Right hemisphere**	
DKI - AK Anterior l.o. Int.caps. (rh)	0.077 ± 0.0012	DKI - AK Ant. l.o.int.caps.	0.056 ± 0.0008	DKI - AK Ant. l.o.int.caps.	0.107 ± 0.0015
DTI - RD Fornix-Striaterminalis (rh)	0.024 ± 0.0008	DTI - FA Superior cerebell.ped.	0.031 ± 0.0004	DTI - RD Fornix-striaterminalis	0.035 ± 0.0007
DTI - FA Superior cerebell. ped. (lh)	0.011 ± 0.0004	DKI - RK Fornix-Striaterminalis	0.022 ± 0.0003	DTI - FA Superior cerebell.ped.	0.034 ± 0.0006
DTI - AD Posterior l.o. ext.caps. (lh)	0.010 ± 0.0003	Cerebellum WM volume	0.022 ± 0.0003	Thalamus volume	0.020 ± 0.0004
Inferior parietal thickness (rh)	0.010 ± 0.0005	Putamen volume	0.019 ± 0.0003	Inferior parietal thickness	0.018 ± 0.0003
Thalamus volume (rh)	0.008 ± 0.0004	Lateral ventricle volume	0.019 ± 0.0003	Cerebellum WM volume	0.018 ± 0.0005
Cerebellum WM volume (rh)	0.007 ± 0.0004	DKI - AK PTR	0.017 ± 0.0003	DKI - AK PTR	0.014 ± 0.0003
DTI - FA Superior cerebell. ped. (rh)	0.007 ± 0.0003	BRIA - vCSF External capsule	0.017	Lateral ventricle volume	0.014 ± 0.0004
BRIA - vCSF External capsule (lh)	0.006 ± 0.0003	WMTI - AWF Superior cerebell.ped.	0.016 ± 0.0002	BRIA - vCSF External capsule	0.013 ± 0.0003
DTI - FA Superior cerebell. ped. (lh)	0.006 ± 0.0002	Thalamus volume	0.016 ± 0.0003	DKI - RK Posterior l.o. inf. ext.caps.	0.012 ± 0.0003
		**diffusion-weighted MRI**			
**Both hemispheres**		**Left hemisphere**		**Right hemisphere**	
DKI - AK Ant. l.o. inf. ext.caps. (rh)	0.129 ± 0.0019	DKI – AK Ant. l.o.int.caps.	0.101 ± 0.0016	DKI – AK Ant. l.o.int.caps.	0.160 ± 0.002
DTI - RD Fornix-Striaterminalis (rh)	0.062 ± 0.0009	DKI – RK Fornix-stria terminalis	0.037 ± 0.0006	DTI – RD Fornix Striaterminalis	0.099 ± 0.0011
DTI - FA Cerebral peduncle (lh)	0.022 ± 0.0004	DTI – FA Superior cerebell.ped.	0.036 ± 0.0005	DKI – AK PTR	0.030 ± 0.0003
DTI - FA Superior cerebell.ped. (lh)	0.017 ± 0.0003	DTI – FA Cerebral peduncle	0.034 ± 0.0006	DTI – FA Superior cerebell.ped.	0.026 ± 0.0003
DTI - AD Posterior l.o. inf. ext.caps. (lh)	0.015 ± 0.0002	BRIA – vCSF ATR	0.027 ± 0.0005	BRIA – vCSF ATR	0.020 ± 0.0004
DKI - AK Ant. l.o. inf. ext.caps. (lh)	0.015 ± 0.0002	DTI – RD Fornix Striaterminalis	0.025 ± 0.0006	DTI – AD CST	0.020 ± 0.0003
DKI - RK Fornix-Striaterminalis (lh)	0.013 ± 0.0003	DKI – AK PTR	0.025 ± 0.0004	DKI – AK SFF	0.020 ± 0.0003
DKI - AK SFF (lh)	0.012 ± 0.0002	DTI – FA IFOF	0.025 ± 0.0004	DKI – AK Ant. corona radiata	0.015 ± 0.0002
DTI - FA IFOF (lh)	0.011 ± 0.0002	WMTI – AWF retroen. l.o. int.caps.	0.023 ± 0.0004	DTI – AD Posterior l.o. int.caps.	0.015 ± 0.0002
DKI - AK PTR (rh)	0.011 ± 0.0002	DKI – AK SFF	0.023 ± 0.0005	BRIA – vCSF SLF	0.013 ± 0.0002
		**T_1-weighted MRI**			
**Both hemispheres**		**Left hemisphere**		**Right hemisphere**	
Inferiorparietal thickness (rh)	0.030 ± 0.0007	Lateral ventricle volume	0.063 ± 0.0008	Lateral ventricle volume	0.097 ± 0.0015
Lateral Ventricle (rh)	0.030 ± 0.0006	Inf.Lat.Vent volume	0.042 ± 0.0009	Inferior parietal thickness	0.044 ± 0.0008
Inf.Lat.Ventricle (lh)	0.026 ± 0.0007	Superior temporal thickness	0.037 ± 0.0011	Superior temporal thickness	0.039 ± 0.0008
Superior temporal thickness (lh)	0.024 ± 0.0007	Putamen volume	0.029 ± 0.0007	Thalamus volume	0.030 ± 0.0008
Putamen volume (lh)	0.020 ± 0.0005	Insula volume	0.029 ± 0.0006	Cerebellum WM volume	0.028 ± 0.0008
Cerebellum WM (rh)	0.017 ± 0.0006	Thalamus volume	0.026 ± 0.0006	Insula volume	0.025 ± 0.0006
Thalamus volume (rh)	0.011 ± 0.0004	Cerebellum WM volume	0.023 ± 0.0007	Inf.Lat.Vent volume	0.021 ± 0.0007
Thalamus volume (lh)	0.011 ± 0.0004	Mean thickness	0.020 ± 0.0005	Inferior temporal area	0.019 ± 0.0004
Superior temporal thickness (rh)	0.011 ± 0.0004	Amygdala volume	0.019 ± 0.0005	Superior temporal thickness	0.018 ± 0.0004
Accumbens area (lh)	0.011 ± 0.0003	Accumbens volume	0.019 ± 0.0005	Temporal pole volume	0.015 ± 0.0005

Supplementary Table 14. Sex stratified brain age model performance

R^2 = Variance explained, MAE = Mean Absolute Error, RMSE = Root Mean Squared Error, Corr. = Correlation, Values in round parentheses () refer to standard deviations and square brackets [] to 95% confidence interval around correlations (Pearson's r) of uncorrected brain age estimates and chronological age. Males' multimodal ($N = 18,876$), T_1-weighted ($N = 23,059$), and dMRI models ($N = 18,950$), and females' multimodal ($N = 20,631$), T_1-weighted ($N = 24,981$), and dMRI models ($N = 20,683$) were estimated.
* The correlation between raw brain age and chronological age.

**Males**					
**Model**	**Features**	$\mathbf{R^2}$	**MAE**	**RMSE**	**Correlation***
Left T_1w	117	0.513 (0.013)	4.398 (0.059)	5.472 (0.087)	0.719 [0.712, 0.725]
Right T_1w	117	0.506 (0.012)	4.437 (0.069)	5.521 (0.101)	0.711 [0.704, 0.717]
T_1w	234	0.534 (0.010)	4.294 (0.070)	5.356 (0.096)	0.722 [0.716, 0.728]
Left dMRI	840	0.573 (0.017)	4.104 (0.077)	5.111 (0.112)	0.761 [0.755, 0.767]
Right dMRI	840	0.586 (0.015)	4.039 (0.063)	5.039 (0.108)	0.767 [0.761, 0.773]
dMRI	1680	0.608 (0.015)	3.922 (0.078)	4.908 (0.108)	0.782 [0.776, 0.787]
Left multimodal	957	0.626 (0.012)	3.794 (0.030)	4.767 (0.037)	0.795 [0.790, 0.801]
Right multimodal	957	0.630 (0.015)	3.783 (0.066)	4.743 (0.075)	0.798 [0.792, 0.803]
Multimodal	1914	0.653 (0.014)	3.688 (0.064)	4.627 (0.040)	0.808 [0.803, 0.813]
**Females**					
**Model**	**Features**	$\mathbf{R^2}$	**MAE**	**RMSE**	**Correlation***
Left T_1w	117	0.482 (0.015)	4.424 (0.053)	5.499 (0.060)	0.696 [0.690, 0.703]
Right T_1w	117	0.470 (0.017)	4.486 (0.07)	5.570 (0.082)	0.688 [0.681, 0.694]
T_1w	234	0.504 (0.015)	4.339 (0.073)	5.403 (0.079)	0.710 [0.704, 0.716]
Left dMRI	840	0.560 (0.014)	4.043 (0.072)	4.993 (0.072)	0.745 [0.739, 0.751]
Right dMRI	840	0.573 (0.014)	3.961 (0.065)	4.925 (0.061)	0.757 [0.751, 0.763]
dMRI	1680	0.597 (0.013)	3.845 (0.069)	4.815 (0.058)	0.773 [0.767, 0.778]
Left multimodal	957	0.608 (0.016)	3.782 (0.016)	4.696 (0.094)	0.778 [0.773, 0.784]
Right multimodal	957	0.613 (0.016)	3.746 (0.095)	4.664 (0.098)	0.785 [0.780, 0.790]
Multimodal	1914	0.633 (0.017)	3.653 (0.085)	4.577 (0.080)	0.798 [0.793, 0.803]

Supplementary Table 15. Tuned hyperparameters for sex stratified brain age models

Overview of the tuned hyperparameters for each of the sex-specific brain age models. Males' multimodal ($N = 18,876$), T_1-weighted ($N = 23,059$), and dMRI models ($N = 18,950$), and females' multimodal ($N = 20,631$), T_1-weighted ($N = 24,981$), and dMRI models ($N = 20,683$) were estimated.

**Males**				
**Modality**	**Hemisphere**	**Learning Rate**	**Maximum Depth**	**Number of Trees**
Multimodal	Both	0.1	6	140
Multimodal	Left	0.1	5	140
Multimodal	Right	0.1	4	180
dMRI	Both	0.1	5	140
dMRI	Left	0.1	4	180
dMRI	Right	0.05	5	180
T_1w	Both	0.1	5	60
T_1w	Left	0.1	4	180
T_1w	Right	0.1	4	180
**Females**				
**Modality**	**Hemisphere**	**Learning Rate**	**Maximum Depth**	**Number of Trees**
Multimodal	Both	0.1	4	180
Multimodal	Left	0.05	8	180
Multimodal	Right	0.05	6	180
dMRI	Both	0.05	7	180
dMRI	Left	0.05	7	140
dMRI	Right	0.05	8	180
T_1w	Both	0.1	5	140
T_1w	Left	0.05	6	180
T_1w	Right	0.1	5	180

SUPPLEMENTARY FIGURES

Supplementary Figure 1. Uncorrected mean values' age curves

Uncorrected standardized and zero-centered age curves and lines for mean values of grey and white and grey matter features by age per hemisphere. For line fitting, first, a cubic smooth function (s) with $k = 4$ knots was applied to plot the relationship between age and brain features (F): $\hat{age} = s(F)$. Second, a linear model was applied of the following form: $\hat{age} = \beta_0 + \beta_1 \times F$. Models used restricted maximum likelihood (REML). Extreme outliers defined by Mean±9SD were removed for visualisation purposes ($N = 35,235$). Source data are provided in Source Data file 14.

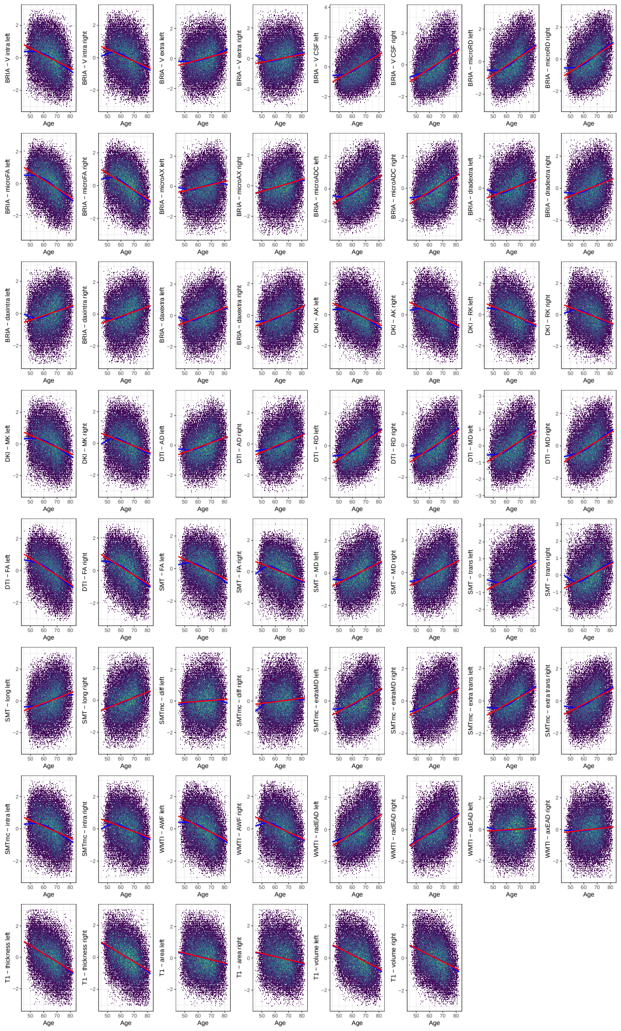

Supplementary Figure 2. Distribution of the *significant and non-significant* slopes of age-related laterality indexed grey and white matter features

We estimated the absolute laterality index ($|LI|$) for each regional feature to assess the overall directionality of asymmetry-age associations. The distributions of age-relationship of $|LI|$ are displayed with the six panels showing the distributions for the modality-specific features (T_1-weighted and diffusion-weighted) for both sexes, males and females. Source data are provided in Source Data file 15.

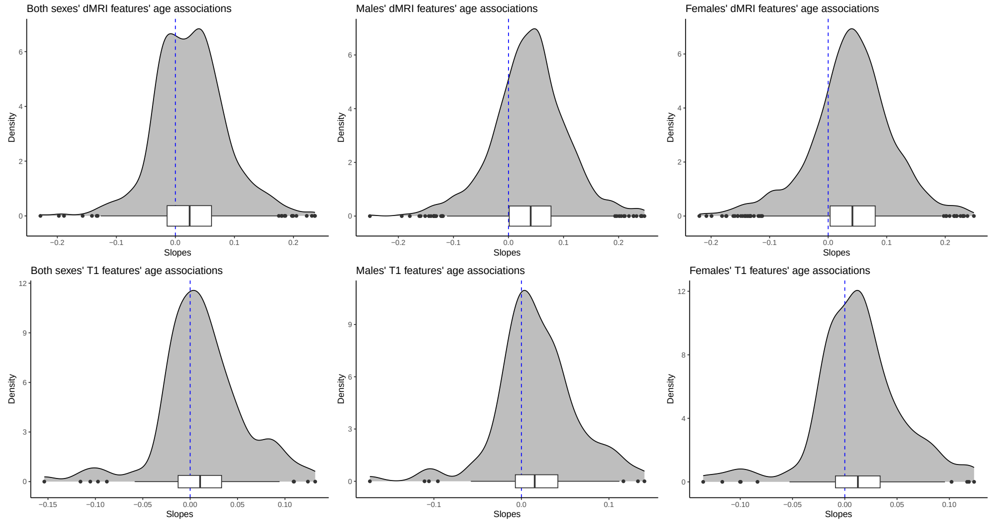

Supplementary Figure 3. Distribution of the *significant* slopes of age-related laterality indexed grey and white matter features

We estimated the absolute laterality index ($|LI|$) for each regional feature to assess the overall directionality of asymmetry-age associations. The distributions of age-relationship of $|LI|$ are displayed with the six panels showing the distributions for the modality-specific features (T_1-weighted and diffusion-weighted) for both sexes, males and females. Source data are provided in Source Data file 15.

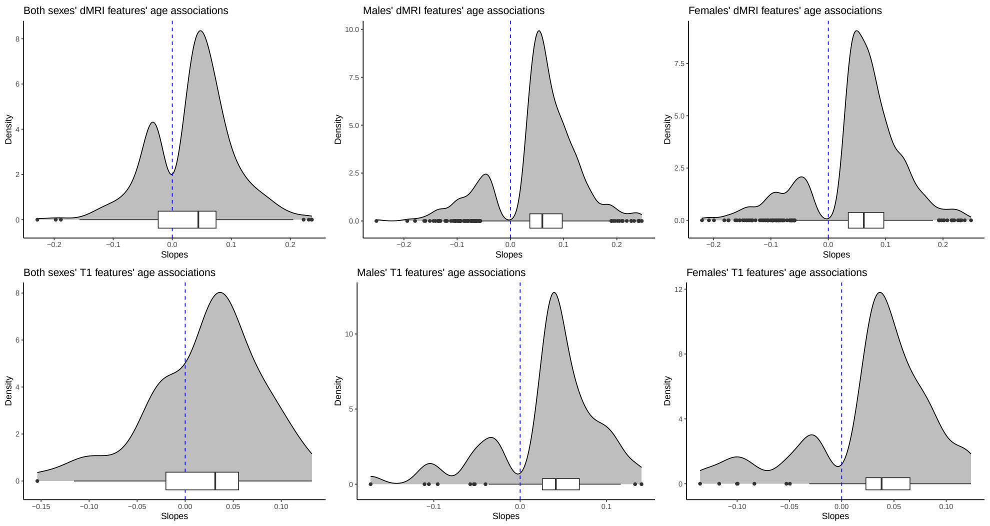

Supplementary Figure 4. Linear, adjusted hemispheric mean values' age associations

Corrected standardized and zero-centered linear age relationships for mean hemispheric values of grey and white matter features by age per hemisphere. Modelling was done using Eq. 2: $a\hat{g}e = \beta_0 + \beta_1 \times F + \beta_2 \times Sex + \beta_3 \times Site$, where F is the respective brain feature. Extreme outliers defined by Mean±9SD were removed for visualisation purposes ($N = 35,235$). Source data are provided in Source Data file 16.

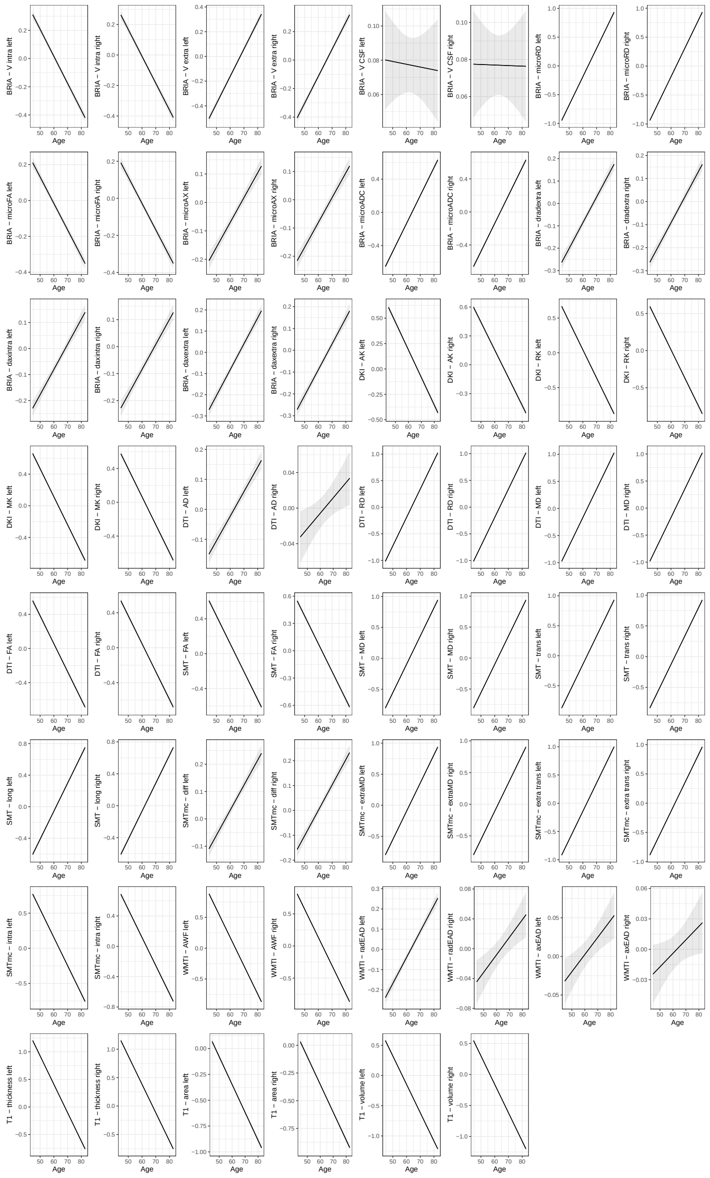

Supplementary Figure 5. Adjusted mean values' hemisphere-specific age associations by sex

Age curves of standardized and zero-centered mean values of GM and WM features per hemisphere and by sex. A cubic smooth function (s) with $k = 4$ knots was applied to plot the relationship between age and brain features correcting for sex and scanner site (F): $\hat{age} = s(F) + sex + site$ using restricted maximum likelihood (REML). The grey shaded area indicates the 95% CI. All age-relationships were significant ($p_{adj} < .05$). Extreme outliers defined by Mean±9SD were removed for visualisation purposes ($N = 35, 235$). Source data are provided in Source Data file 17.

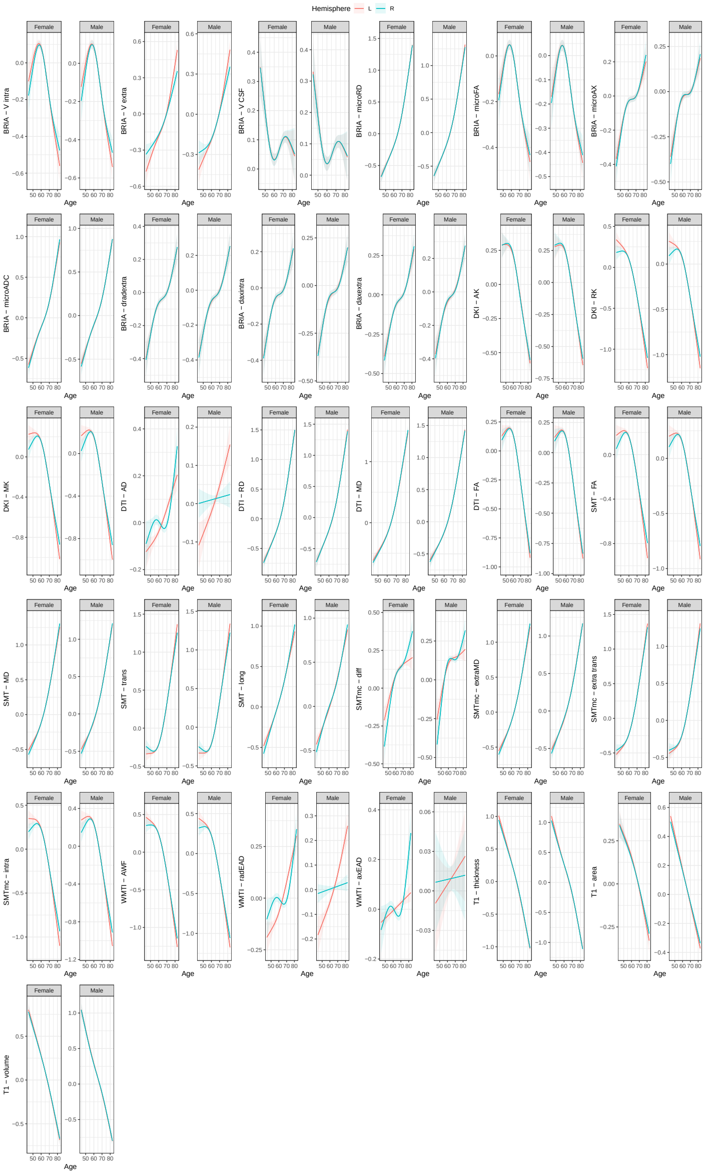

Supplementary Figure 6. Adjusted mean values' sex-specific age associations by hemisphere

Age curves of standardized and zero-centered mean values of GM and WM features per hemisphere and by sex. A cubic smooth function (s) with $k = 4$ knots was applied to plot the relationship between age and brain features correcting for sex and scanner site (F): $\hat{age} = s(F) + sex + site$ using restricted maximum likelihood (REML). The grey shaded area indicates the 95% CI. All age-relationships were significant ($p_{adj} < .05$). Extreme outliers defined by Mean±9SD were removed for visualisation purposes ($N = 35,235$). Source data are provided in Source Data file 17.

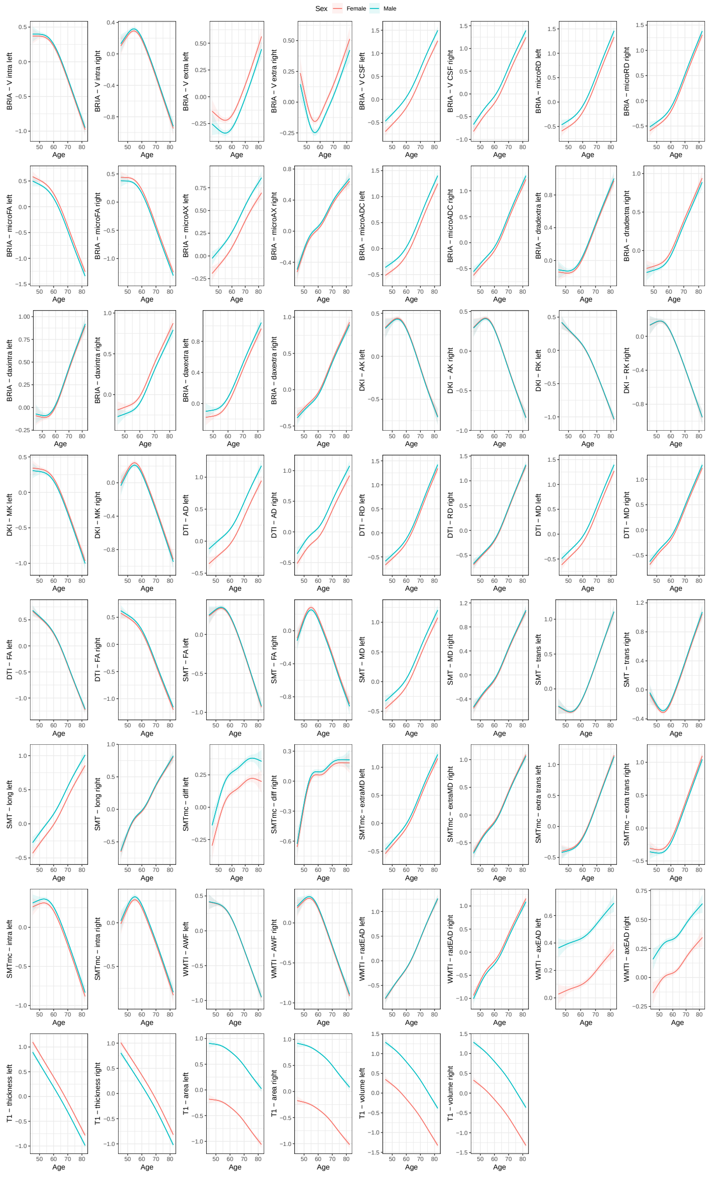

Supplementary Figure 7. Association between general health-and-lifestyle phenotypes and brain age estimated from different modalities, left, right and both hemispheres by sex

Eq. 9 was used (yet stratifying by sex) and standardized slopes are presented. For simplicity, standardized slopes with $|\beta| < 0.005$ were rounded down to $\beta = 0$. Panel a) males ($N = 16,765$), panel b) females ($N = 18,900$). Missingness in predictors for females: $N_{Alcohol} = 121$, $N_{BMI} = 695$, $N_{Diastolic\ Blood\ Pressure} = 2,454$, $N_{Height} = 662$, $N_{Hip\ Circumference} = 635$, $N_{Pulse\ Pressure} = 2,454$, $N_{Smoking} = 192$, $N_{Smoking\ Status} = 117$, $N_{Systolic\ Blood\ Pressure} = 2,454$, $N_{Waist\ Circumference} = 636$, $N_{Weight} = 668$, $N_{WHR} = 636$. Missingness in predictors for males: $N_{Alcohol} = 116$, $N_{BMI} = 552$, $N_{Diastolic\ Blood\ Pressure} = 1,988$, $N_{Height} = 527$, $N_{Hip\ Circumference} = 518$, $N_{Pulse\ Pressure} = 1,988$, $N_{Smoking} = 147$, $N_{Smoking\ Status} = 107$, $N_{Systolic\ Blood\ Pressure} = 1,988$, $N_{Waist\ Circumference} = 518$, $N_{Weight} = 544$, $N_{WHR} = 518$. L: left hemisphere, R: right hemisphere, LR: both hemispheres, BMI: body mass index, WHR: waist-to-hip ratio. Bonferroni-adjusted $p < .05$ is marked by a black frame. Source data are provided in Source Data file 18.

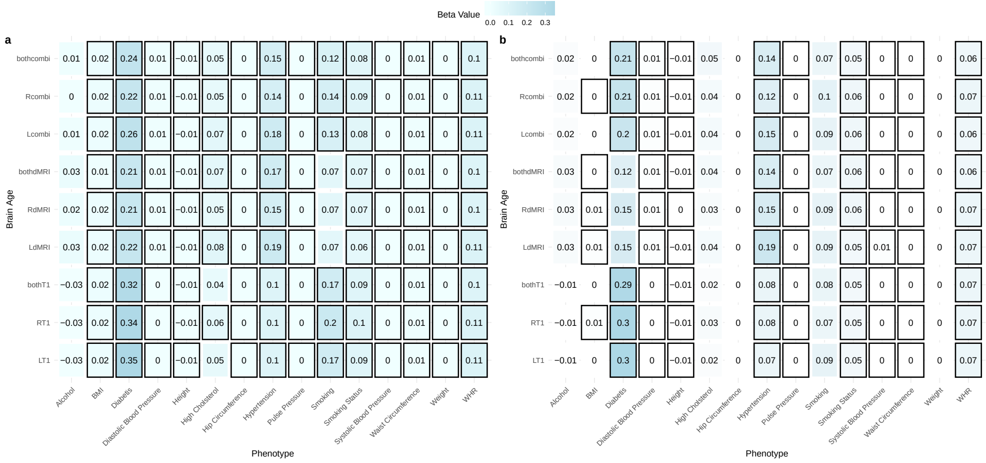

Supplementary Figure 8. Males' T_1-weighted and dMRI features asymmetry-age-associations

T_1-weighted and dMRI features linear asymmetry-age-associations. The plot presents the standardized, site-corrected regression slopes versus Bonferroni-adjusted -log10 p-values for males. Modelling was done using a sex-stratified version of Eq. 2: $a\hat{g}e = \beta_0 + \beta_1 \times F + \beta_2 \times Site$, where F is the respective brain feature. Labelling was done separately for T_1-weighted and dMRI indicating the 10 most significantly associated features (five for $\beta > 0$ and five for $\beta < 0$). Cereb.Peduncle = cerebral peduncle, Rostro-mid. thicknes = rostro-middle thickness, SLFT = superior longitudinal fasciculus (temporal part), Fornix-Str.Term. = fornix-stria terminalis tract, Rost. ant. cingulate = rostral anterior cingulate. $N = 18,876$. Source data are provided in Source Data file 19.

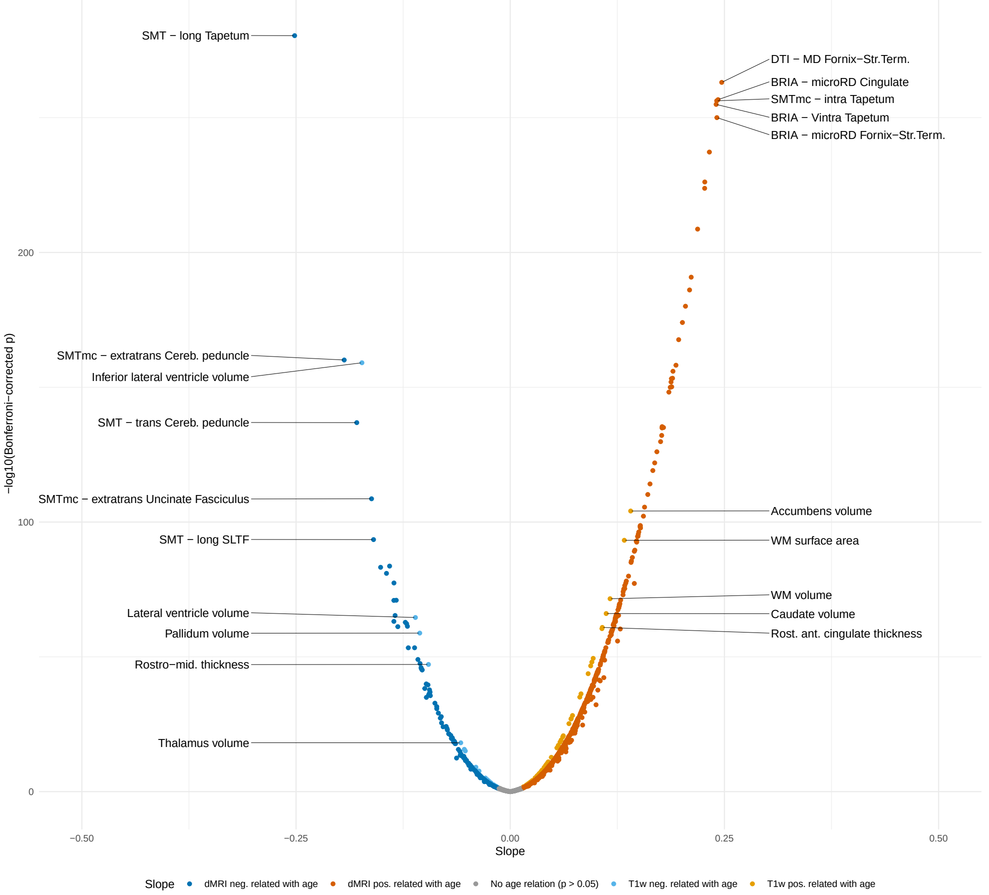

Supplementary Figure 9. Females' T_1-weighted and dMRI features asymmetry-age-associations

T_1-weighted and dMRI features linear asymmetry-age-associations. The plot presents the standardized, site-corrected regression slopes versus Bonferroni-adjusted -log10 p-values for females. Modelling was done using a sex-stratified version of Eq. 2: $\hat{age} = \beta_0 + \beta_1 \times F + \beta_2 \times Site$, where F is the respective brain feature. Labelling was done separately for T_1-weighted and dMRI indicating the 10 most significantly associated features (five for $\beta > 0$ and five for $\beta < 0$). Cereb.Peduncle = cerebral peduncle, Rostro-mid. thicknes = rostro-middle thickness, SLFL = superior longitudinal fasciculus, Sup.front.occ.Fasc. = superior fronto-occipital fasciculus. $N = 20,631$. Source data are provided in Source Data file 19.

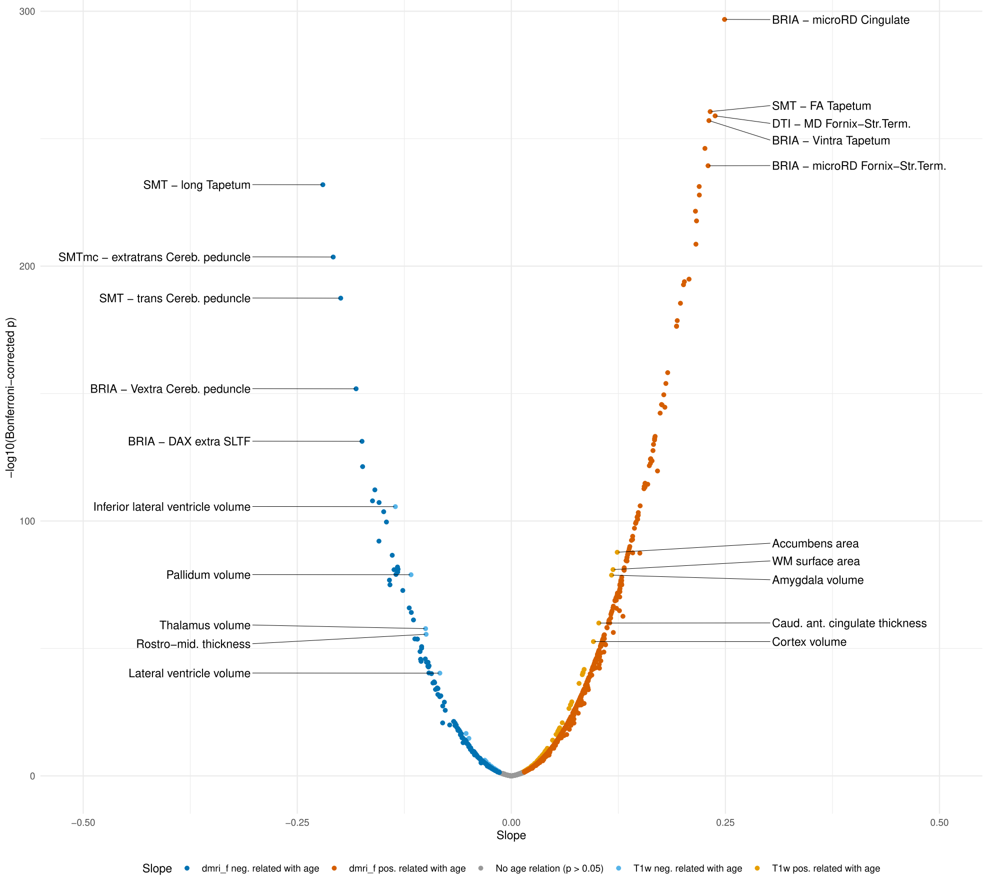

Supplementary Figure 10. Pearson correlation coefficients between chronological and predicted ages for T_1-weighted, diffusion, and multimodal MRI for left, right and both hemispheres for sex stratified brain age models

All Bonferroni-corrected $p < .001$. L: left hemisphere, R: right hemisphere, LR: both hemispheres. Left panel: males ($N = 16,765$), right panel: females ($N = 18,900$). Source data are provided in Source Data file 20.

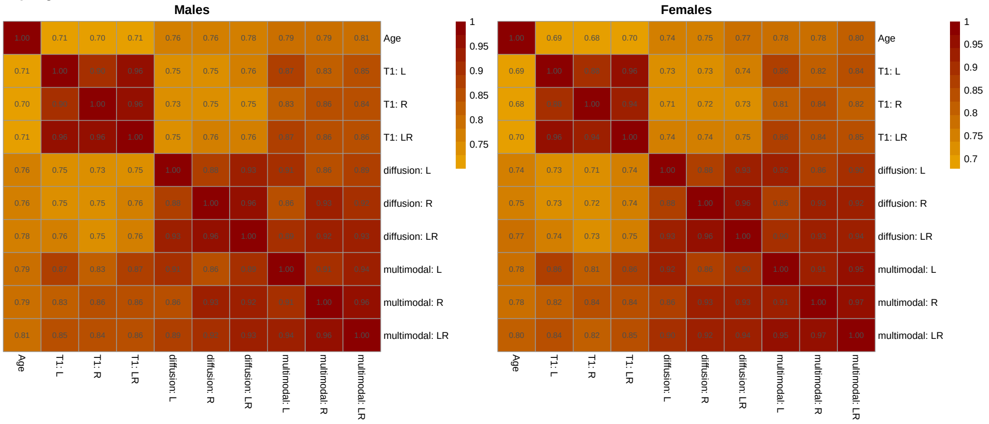

Supplementary Figure 11. Association between general health-and-lifestyle phenotypes and sex-specific trained brain age estimated from different modalities, left, right and both hemispheres

Eq. 9 was used (yet stratifying by sex) and standardized slopes are presented. For brain age prediction, we used models which were trained separately for males and females, respectively. For simplicity, standardized slopes with $|\beta| < 0.005$ were rounded down to $\beta = 0$. L: left hemisphere, R: right hemisphere, LR: both hemispheres, BMI: body mass index, WHR: waist-to-hip ratio. Bonferroni-adjusted $p < .05$ is marked by a black frame. Sample size: $N = 35,665$. Missingness in predictors: $N_{Alcohol} = 237$, $N_{BMI} = 1,247$, $N_{Diastolic\ Blood\ Pressure} = 4,442$, $N_{Height} = 1,189$, $N_{Hip\ Circumference} = 1,153$, $N_{Pulse\ Pressure} = 4,442$, $N_{Smoking} = 339$, $N_{Smoking\ Status} = 224$, $N_{Systolic\ Blood\ Pressure} = 4,442$, $N_{Waist\ Circumference} = 1,154$, $N_{Weight} = 1,212$, $N_{WHR} = 1,154$. Source data are provided in Source Data file 21.

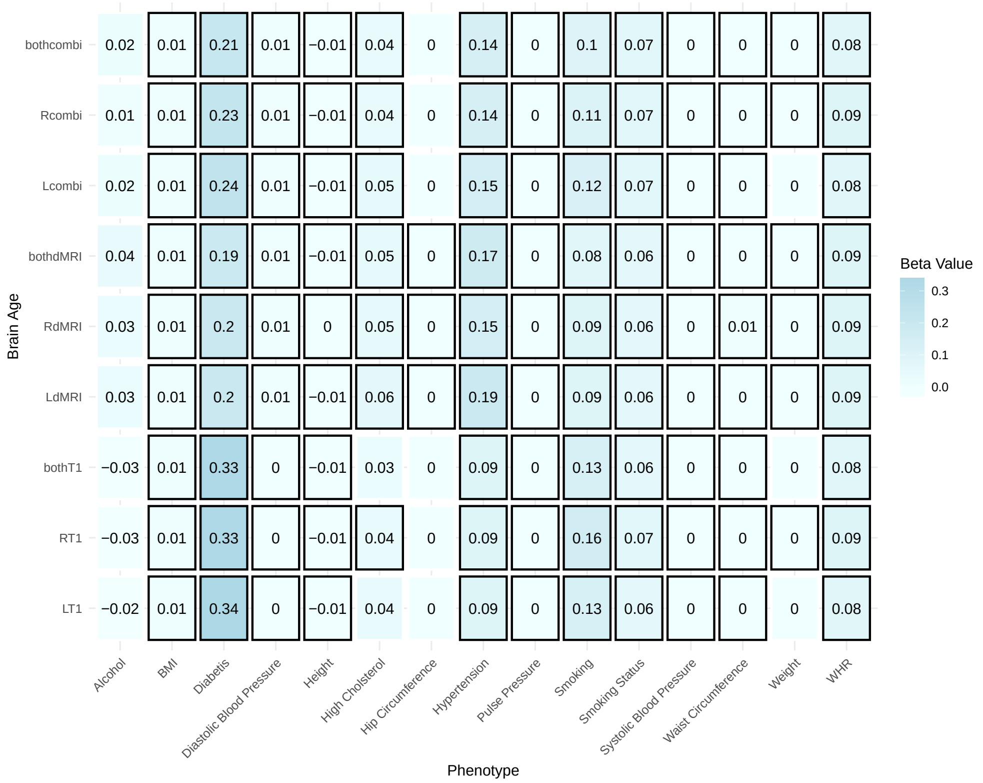

Supplementary Figure 12. Association between general health-and-lifestyle phenotypes and sex-specific trained brain age estimated from different modalities, left, right and both hemispheres by sex

Eq. 9 was used (yet stratifying by sex) and standardized slopes are presented. For brain age prediction, we used models which were trained separately for males ($N = 16,765$) and females ($N = 18,900$), respectively. Missingness in predictors for females: $N_{Alcohol} = 121$, $N_{BMI} = 695$, $N_{Diastolic\ Blood\ Pressure} = 2,454$, $N_{Height} = 662$, $N_{Hip\ Circumference} = 635$, $N_{Pulse\ Pressure} = 2,454$, $N_{Smoking} = 192$, $N_{Smoking\ Status} = 117$, $N_{Systolic\ Blood\ Pressure} = 2,454$, $N_{Waist\ Circumference} = 636$, $N_{Weight} = 668$, $N_{WHR} = 636$. Missingness in predictors for males: $N_{Alcohol} = 116$, $N_{BMI} = 552$, $N_{Diastolic\ Blood\ Pressure} = 1,988$, $N_{Height} = 527$, $N_{Hip\ Circumference} = 518$, $N_{Pulse\ Pressure} = 1,988$, $N_{Smoking} = 147$, $N_{Smoking\ Status} = 107$, $N_{Systolic\ Blood\ Pressure} = 1,988$, $N_{Waist\ Circumference} = 518$, $N_{Weight} = 544$, $N_{WHR} = 518$. For simplicity, standardized slopes with $|\beta| < 0.005$ were rounded down to $\beta = 0$. Panel a) males, panel b) females. L: left hemisphere, R: right hemisphere, LR: both hemispheres, BMI: body mass index, WHR: waist-to-hip ratio. Bonferroni-adjusted $p < .05$ is marked by a black frame. Source data are provided in Source Data file 22.

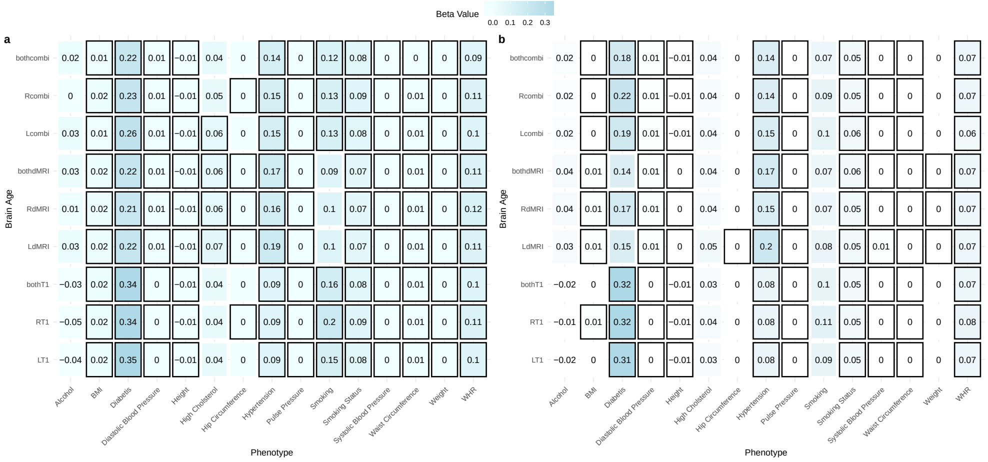

Supplementary Note - Recap of the Statistical Analyses

All statistical analyses were carried out using Python (v3.7.1) and R (v4.2.0).

Hemispheric differences and age sensitivity

To give an overview of the extent of brain asymmetry, we assessed the significance of T_1-weighted and dMRI features' asymmetry using two-sided t-tests. The lateralisation or asymmetry of the brain features was estimated as the following: we applied the LI[7] to both regional features and features averaged over each hemisphere (see also[8]).

$$LI = \frac{L - R}{L + R}, \tag{1}$$

where L and R belongs to any left and right scalar metric, respectively. Furthermore, when associating LI with age, we used absolute LI values ($|LI|$) allowing to estimate age-effects on asymmetry irrespective of the direction of the asymmetry (leftwards or rightwards).

We then used linear regression models correcting for sex and scanning site to predict age from all regular and LI features:

$$\hat{Age} = F + Sex + Site, \tag{2}$$

where F is a scalar metric such as, for example, hippocampus volume (derived from T_1-weighted image) or tapetum fractional anisotropy (derived from DTI). The same model setup was used applying generalised additive models (GAM) to model non-linear relationships between F and Age using a smooth s of linked quadratic functions with k = 4 knots and restricted maximum likelihood (REML):

$$\hat{Age} = s(F) + Sex + Site. \tag{3}$$

Likelihood ratio tests (LRTs)[9] were used to assess the age sensitivity of all T_1-weighted and dMRI features and their asymmetry/LI features by comparing the above models with baseline models not including the respective feature:

$$\hat{Age} = Sex + Site. \tag{4}$$

We used the same procedure for region-averaged and hemispheric average metrics for regular and LI features. Hemispheric averages of regular features were then visualised by age, including surface area, volume, thickness for T_1-weighted data, and intra- and extra-axonal water diffusivities as well as for DTI and DKI metrics.

To compare the model fit of non-linear and linear models we used the Akaike information criterion (AIC)[10] and Bayesian information criterion (BIC)[11].

Brain age assessment

We estimated correlations across HBA and GBA to assess their similarities in addition to the model output provided from the prediction procedure. We also correlated age with the LI (see Eq. 1) for the three modalities (dMRI, T_1-weighted, multimodal MRI), and estimated the age sensitivity of the LI as described in (Eqs. 2-4).

As preregistered (https://aspredicted.org/if5yr.pdf), to test the relationships between hemisphere (H), modality (M), and HBA while controlling for age, sex and scanner site, we employed linear mixed effects regression (LMER) models of the following form:

$$H\hat{B}A = H + M + H \times M + Sex + Age + Sex \times Age + (1|Site) + (1|I), \tag{5}$$

where I refers to the random intercept at the level of the individual. Post-hoc group differences were observed for hemisphere, modality and their interaction.

Next, handedness (Ha) was added to the model to observe whether there are model differences between the resulting LMER:

$$H\hat{B}A = Ha + H \times Ha + H + M + H \times M + Sex + Age + Sex \times Age + (1|Site) + (1|I), \tag{6}$$

and the previous model. Models were statistically compared using LRTs[9].

For sex-stratified analyses, we considered brain age estimates both from models using data from both sexes together, as well as models which were trained on females-only or males-only data. The modelling choice (MC) was included as a factor for the sex-stratified brain age analyses in the formula of Eq. 6.

Finally, the LIs (Eq. 1 of left and right brain age predictions for T_1-weighted, diffusion and multimodal MRI (LI_{HBA}, i.e. the asymmetry in brain age predictions) were associated with age, controlling for sex and scanner site as random effect:

$$\hat{Age} = LI_{HBA} + Sex + (1|Site). \tag{7}$$

The LI_{HBA}s' age-sensitivity was then assessed (as for brain features, see Eqs. 2-4), using LRTs comparing the above model with a baseline model excluding LI_{HBA} (Eq. 4):

$$\hat{Age} = Sex + (1|Site). \tag{8}$$

This procedure was also done for each sex individually, also separating between brain age models predictions which were obtained from the data from both sexes compared to a single sex.

Phenotype associations of brain age

In an exploratory analysis step, we assessed association patterns between brain ages and health-and-lifestyle factors which have previously demonstrated an association with brain age[12–17]. This analysis step served to compare phenotype associations across estimated brain ages. The health-and-lifestyle factors included alcohol drinking (binary), height and weight supplementing body mass index (BMI), diabetes diagnosis (binary), diastolic blood pressure, systolic blood pressure, pulse pressure, hypertension (binary), cholesterol level (binary), and smoking (binary describing current smokers). For this last analysis step, LMERs were used with the following structure:

$$\hat{P} = BA + Sex + Age + Sex \times Age + (1|Site), \quad (9)$$

where BA refers brain age incorporating both GBA and HBA, P is the phenotype.

Furthermore, where applicable, we corrected p-values for multiple testing using Bonferroni correction and an α-level of $p < .05$. This involves multiplying the p-value by the number of tests used to test the same hypothesis. Adjusted p-values are marked as p_{adj} and unadjusted p-values as p. We used a high-precision approach to calculate exact p-values utilising the Multiple Precision Floating-Point Reliable R package[18], and report standardised β-values. Sex and site were entered as independent factorial nominal variables in the applicable regression models, with sex being a binary (0 = female, 1 = male) and scanner site a multinominal (0 = Cheadle, 1 = Newcastle, 2 = Reading, 3 = Bristol). Finally, we repeated the presented statistical analyses stratifying for sex.

REFERENCES

[1] Reisert, M., Kellner, E., Dhital, B., Hennig, J. & Kiselev, V. G. Disentangling micro from mesostructure by diffusion MRI: a Bayesian approach. *NeuroImage* **147**, 964–975 (2017).

[2] Jensen, J. H., Helpern, J. A., Ramani, A., Lu, H. & Kaczynski, K. Diffusional kurtosis imaging: the quantification of non-gaussian water diffusion by means of magnetic resonance imaging. *MR in Med.* **53**, 1432–1440 (2005).

[3] Fieremans, E., Jensen, J. H. & Helpern, J. A. White matter characterization with diffusional kurtosis imaging. *NeuroImage* **58**, 177–188 (2011).

[4] Basser, P. J., Mattiello, J. & LeBihan, D. Mr diffusion tensor spectroscopy and imaging. *Biophys. J.* **66**, 259–267 (1994).

[5] Kaden, E., Kruggel, F. & Alexander, D. C. Quantitative mapping of the per-axon diffusion coefficients in brain white matter. *MR in Med.* **75**, 1752–1763 (2016).

[6] Kaden, E., Kelm, N. D., Carson, R. P., Does, M. D. & Alexander, D. C. Multi-compartment microscopic diffusion imaging. *NeuroImage* **139**, 346–359 (2016).

[7] Benson, R. *et al.* Language dominance determined by whole brain functional mri in patients with brain lesions. *Neurol.* **52**, 798–798 (1999).

[8] Williams, C. M., Peyre, H., Toro, R. & Ramus, F. Comparing brain asymmetries independently of brain size. *NeuroImage* **254**, 119118 (2022).

[9] Lehmann, E. L. On likelihood ratio tests. *Selected works of E.L. Lehmann* 209–216 (2012).

[10] Akaike, H. in *Information theory and an extension of the maximum likelihood principle* 199–213 (Springer, 1998).

[11] Neath, A. A. & Cavanaugh, J. E. The bayesian information criterion: background, derivation, and applications. *Wiley Interdisc. Rev.: Comp. Stat.* **4**, 199–203 (2012).

[12] Beck, D. *et al.* Adipose tissue distribution from body MRI is associated with cross-sectional and longitudinal brain age in adults. *NeuroImage: Clin.* **33**, 102949 (2022).

[13] Beck, D. *et al.* Cardiometabolic risk factors associated with brain age and accelerate brain ageing. *HBM* **43**, 700–720 (2022).

[14] Korbmacher, M. *et al.* Bio-psycho-social factors' associations with brain age: a large-scale uk biobank diffusion study of 35,749 participants. *Front. Psych.* **14**, 1117732 (2023).

[15] Leonardsen, E. H. *et al.* Deep neural networks learn general and clinically relevant representations of the ageing brain. *NeuroImage* **256**, 119210 (2022).

[16] Cole, J. H. Multimodality neuroimaging brain-age in uk biobank: relationship to biomedical, lifestyle, and cognitive factors. *Neurobio. Aging* **92**, 34–42 (2020).

[17] Smith, S. M. *et al.* Brain aging comprises many modes of structural and functional change with distinct genetic and biophysical associations. *eLife* **9**, e52677 (2020).

[18] Maechler, M., Maechler, M. M., MPFR, S., Suggests, M. & SuggestsNote, M. Package 'rmpfr'. *CRAN* (2016). URL https://cran.r-project.org/web/packages/Rmpfr/index.html.
